# Perceiving amputee gait from biological motion: kinematics cues and effect of experience level

**DOI:** 10.1038/s41598-020-73838-y

**Published:** 2020-10-13

**Authors:** I.-Chieh Lee, Matheus M. Pacheco, Michael D. Lewek, He Huang

**Affiliations:** 1 UNC-NC State Joint Department of Biomedical Engineering, North Carolina State University and University of North Carolina at Chapel Hill, 1407 - Engineering Building III, 1840 Entrepreneur Drive, Raleigh, NC 27695 USA; 2 School of Physical Education and Sport, University of São Paulo, São Paulo, Brazil; 3 Division of Physical Therapy, Department of Allied Health Sciences, University of North Carolina at Chapel Hill, Chapel Hill, NC USA

**Keywords:** Motion detection, Pattern vision

## Abstract

Physical therapists (PT) and clinicians must be skilled in identifying gait features through observation to assess motor deficits in patients and intervene appropriately. Inconsistent results in the literature have led researchers to question how clinical experience influences PT’s gait perception and to seek the key kinematic features that should be trained to enhance PT’s skill. Thus, this study investigated (1) what are the informative kinematic features that allow gait-deviation perception in amputee gait and (2) whether there are differences in observational gait skills between PT and individuals with less clinical experience (PT students [PTS] and Novices). We introduced a new method that combines biological motion and principal component analysis to gradually mesh amputee and typical walking patterns. Our analysis showed that on average the accuracy rate in identifying gait deviations between PT and PTS was similar and better than Novices. Also, we found that PT’s experience was demonstrated by their better perception of gait asymmetry. The extracted principal components demonstrated that the major gait deviation of amputees was the medial–lateral body sway and spatial gait asymmetry.

## Introduction

Perception of intentions, emotions and actions through the movement of others is an important skill that individuals use to aid our own survival and the wellbeing of all in a social group. It is impressive that by just observing someone’s walking pattern, we can recognize the emotional status, gender, and health condition of the walker and, even, identify a known individual^1,2^. This is observed even when much of the information such as the face, clothes, or hairstyle is obscured. One explanation for such perceptual ability is that there is critical information that is distributed, and therefore perceivable, through movement; information that is kinematically invariant^2,3^.

This ability to analyze gait patterns is clinically relevant as, through observation, physical therapists (PT) and other clinicians can assess motor deficits in patients^4^. Such visual assessment has been widely used in rehabilitation because of rapidity, simplicity and economy. Observational gait analysis (OGA) can provide an overall assessment of an individual’s gait pattern and helps to determine gross abnormalities that may exist^5^. Since accuracy on OGA requires practice, this skill is a focus of PT education. Thus, it is expected that through training and experience, PT learn to attend to the most useful information allowing them to judge gait accurately.

Several studies have investigated this skill by asking PT and other clinicians to judge gait impairment by watching patients walking in situ or through videotapes. The results show that PT and other clinicians’ judgments vary from inaccurate^6^, reasonably accurate^7^, to accurate^8,9^. Studies using slow-motion^10^ or freeze-frame videotapes^11^ found limited inter- and intra-rater reliability (see also^4,12^). Given the lack of a standardized training or assessment protocol to ensure the accuracy of OGA^10^, including the inconsistent results in the literature, we question whether clinicians exhibit better perception of gait patterns than untrained individuals.

The explanation for the disparate results could be due to the methods used for evaluating OGA, such as the methods of displaying gait^8,11,13^, condition of patients^6,9,10^, and the use of an analysis form^14,15^. Hughes and Bell^11^ attributed the low rate in identifying gait asymmetry to the change of viewing angle as participants walked toward and away from the camera. Eastlack, Arvidson, Snyder-Mackler, Danoff and McGarvey^10^ suspected that structural deformities and abnormalities in the gait pattern (e.g., inadequate knee genu valgus/varus) could mislead the rater in judging step length. Importantly, the lack of control in the experimental design also limits the possibility of understanding the specific gait features that PT use to judge. This is of relevance; if gait features perceived by PT are found to be useful, these should be goals for intervention, else, we understand why clinical observation is inaccurate. Whereas many studies recognize the inconsistency in the literature, there has been no attempt to accommodate these inconsistencies or discern the perceivable gait features that clinicians attend to. Therefore, we have no firm stance on the question of whether clinicians are accurate and reliable on gait judgement and which gait features would base accurate judgements.

There are two common paradigms employed in finding gait features: biomechanical analyses^16,17^ and rating through OGA^6,14,15^. Biomechanical analyses are helpful to identify potential parameters of the movement that differentiate the gait pattern between patients and unimpaired walkers. However, the variables identified in these studies (e.g., step length, step height, peak force) cannot be directly regarded as the PT perceived variables in their visual gait examination, since the found differences might be too small to be perceived^6^. OGA is well suited to provide clues about gait perception given it is directly based on judgements^6,8,13^. Nevertheless, OGA is performed in a context with a possible range of confounding information. Redundant information such as a grimace of pain, body figure (e.g., large body mass) or stereotype of certain gait dysfunction (e.g., wearing a prosthesis) might confound experimenters on the features that raters might be attending.

A method to guarantee that individuals can only attend to kinematic features is to isolate the biological motion. Johansson^18^ showed that a video of a small number of dots moving, representing the major joints of a walker. The motions of the dots contain information of the global shape and motion. In addition, the relative motion among all local individual dots results in a vivid perception of a human walking. This point-light display is designed to separate biological motion information from other sources of information that are normally intermingled. Many studies have since used this technique to investigate the critical information that is available throughout the movement^2,3^. Such information is kinematically invariant, not affected by the underlying structural difference in walkers (e.g., gait variability or body figure). It has been demonstrated that point-light displays contain enough information for recognition of gender^19,20^, emotional status^21^, and the identity of the walker—perceivers can even determine whether the pattern refers to themselves^22,23^. The phenomena of biological motion perception can be explained by the ability of the human visual system to perceptually organize the individual moving dots into the coherent shape of an articulated human body. Hence, we expect that it can serve as a tool to reveal the capacity of PT to judge gait abnormality and to infer the critical gait features that PT use to form their judgment.

We acknowledge that OGA also encompass the interpretation of the observed movement pattern linking it to a cause (diagnosis), and proposing effective interventions to improve the pattern (treatment). However, PTs capacity to differentiate the pattern from normality is the 1st step of OGA. Thus, a valid diagnosis has to be based on an accurate perception of the observed gait. We therefore focused this paper on the initial part of OGA—perception of amputee gait deviations.

In this study, we investigated (1) if clinical experience influences how PT perceive gait, and (2) the kinematic features PT employ to differentiate amputee gait patterns from typical walking. In order to achieve our goals, we utilized the principal components (through principal component analysis, PCA) of amputees and a control individual walking pattern to develop the biological motion videos.

Principal component analysis (PCA) is a technique for reducing the dimensionality of data set, increasing interpretability but at the same time minimizing information loss. The method finds, through the covariance matrix, the relation between variables and it extracts them as uncorrelated variables from the original data set. From this new set of variables (principal components—PCs), we can utilize the ones that capture a high percentage of variance from the original data set, preserving a great amount of the original structure. This method have been applied in studying coordination of human walking, identifying normal gait features in healthy young adults24,25 and gait disorders (i.e., knee osteoarthritis26, stroke27, Parkinson’s disease28). Here, we used PCA to extract the spatial–temporal features of amputees and, synthesizing it in varying scale with a typical walking, we evaluated PT, PTS and Novice raters in their ability to differentiate from most to least abnormal patterns of gait.

The joint trajectories of three amputee walkers [Walker A (K3-level), Walker B (K4-level) and Walker C (K4-level)] and a control walker were utilized to generate three sets of testing videos. These videos were built to present a range of scaled gait abnormalities. Each set of testing videos contained six biological motion videos generated from one of the amputees, and the biological motion within the six videos linearly scaled from 0 to 100% (in steps of 20%) from the control walker to the amputee walking pattern (for an example, see Movie A). The level of gait abnormality within the 6 videos was varied from most abnormal (the amputee gait) to most normal (control walker’s gait) for raters to sequence. The 3 amputees were treated as independent variables/conditions in this study. We selected one K3 and two K4 amputees in order to have two levels of gait abnormality within the generated videos—large differences between K3 and K4 and small differences within the two K4 amputees. This was meant to better identify experience differences between the groups.

**Figure 1:**
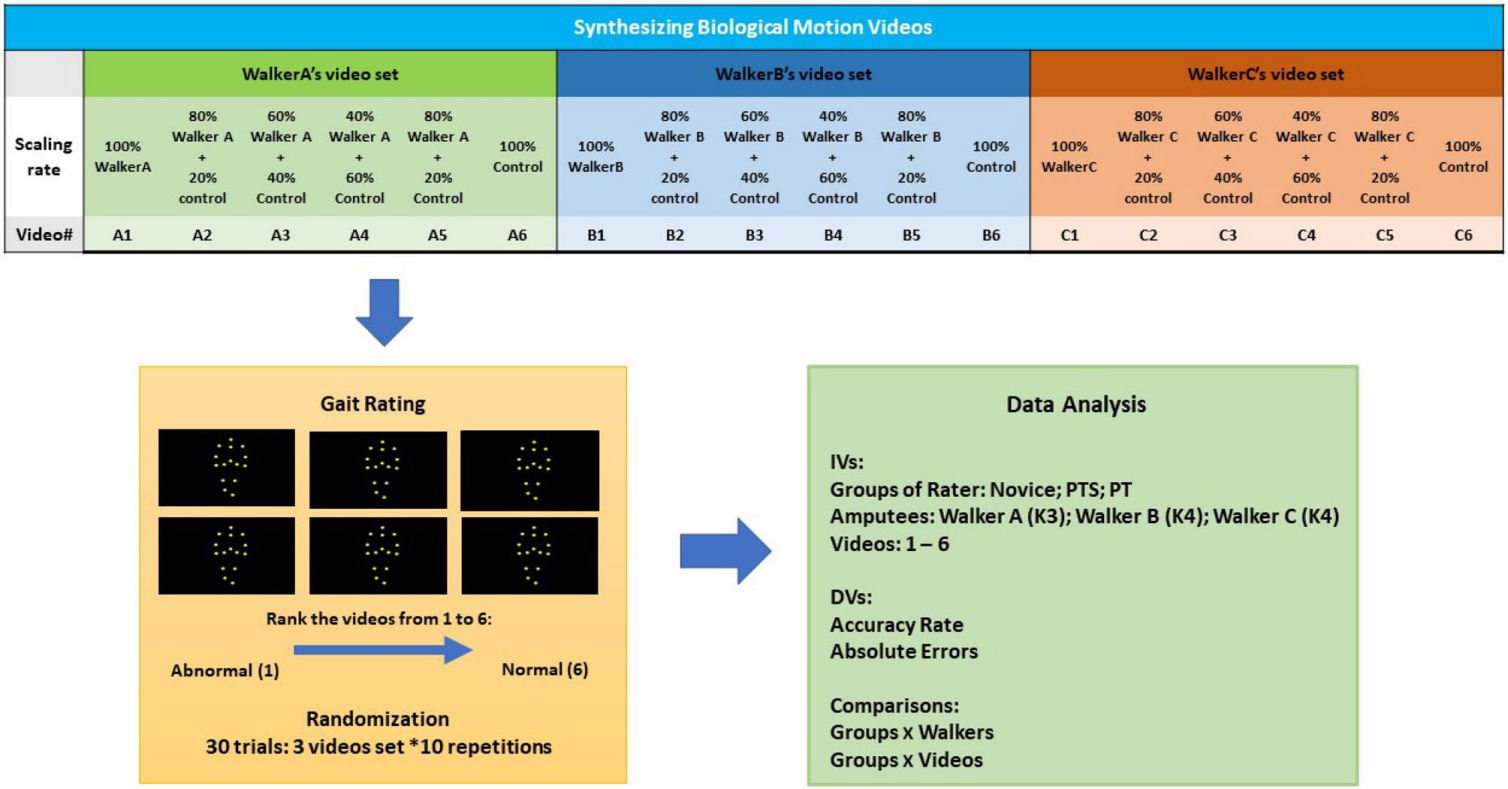
Scheme of the study design.

Then, we tested the accuracy in judging gait of three group of raters (10 PT, 10 PT students and 10 Novice). For each trial, one set of videos were simultaneously presented to the rater to rank the videos from 1 to 6 based on their perceived gait abnormality (1-most abnormal, 6-most normal). The three sets of videos were tested and presented for 10 times in a random order (30 trials: 3 video sets*10 repetitions). We analyzed each rater’s (1) accuracy rate and (2) absolute error in judging each Walker’s videos to estimate the participants abilities in judging walking patterns (see Fig. 1 for the scheme of study design).

We quantified the gait pattern difference between amputee walkers and the control walker based on the extracted movement features from PCA. For each amputee, the extracted features were categorized into similar or dissimilar PCs to the control walker using normalized dot product. The percentage of the gait similarity to the control walker, the degree of gait symmetry angle and magnitude of joints trajectories variance were utilized to capture the main differences between amputees and control walker.

We hypothesized that the accuracy rate in judging videos would be associated with the level of clinical experience; specifically, PT would be better than PT Students and PT Students would be better than Novice. Moreover, we expected that PT would be more sensitive in differentiating the less pronounced kinematics features demonstrated on the amputee gait given their expertise. Besides, the amputee, whose video demonstrates large gait deviations from control walker, would be easier to be distinguished and this would be identifiable through the PCA features extracted by our method.

## Results

### Judgements of videos

#### Accuracy rate

**Figure 2:**
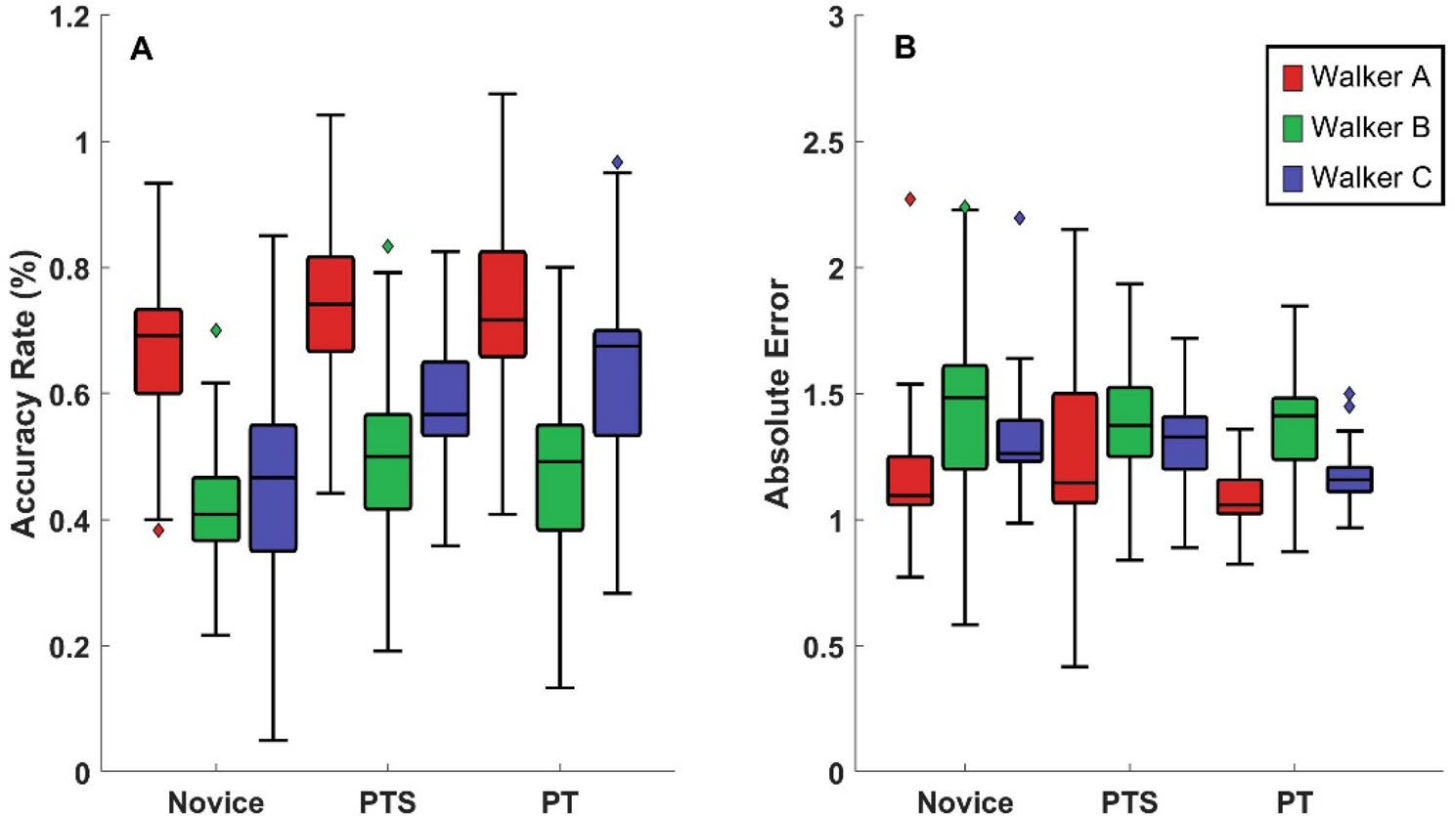
Box-and-whisker plot for judgements of videos. All outliers are lying below or above 1st (Q1) and 3rd quartile (Q3). The maximum and minimum ranges are 1.5 interquartile range above Q3 and below Q1. The results of Walker A, Walker B and Walker C are showed with red, green and blue color, respectively. (**A**) Accuracy rate of each group judging amputee walkers is plotted. (**B**) Absolute error between the rater’s judgment and the correct answer of each groups judging amputee walkers is plotted.

Figure 2A shows the box-and-whisker plot of the accuracy rate for each group of raters in terms of each video set (three amputee walkers). The comparison between groups on accuracy rate showed that Novices were less accurate than PT and PTS and there was no difference between PT and PTS. Walkers’ comparison showed rating Walker A’s video set to be the one which all raters were more accurate and Walker B’s videos set was the least accurate. The permutation test indicated main effects for groups (*p* = 0.003) and walkers (*p* = 0.001), and no significant interaction between walkers and groups (*p* = 0.541). The pairwise comparison between groups showed that the Novice group was less accurate than PT (*p* = 0.016) and PTS (*p* = 0.027) with no significant differences between PT and PTS. Comparison between walkers showed that all groups rated Walker A video set more accurately than Walker B (*p* = 0.001) and C (*p* = 0.002) video sets. Also, Walker C video set was rated more accurately than Walker B video set (*p* = 0.040).

**Figure 3:**
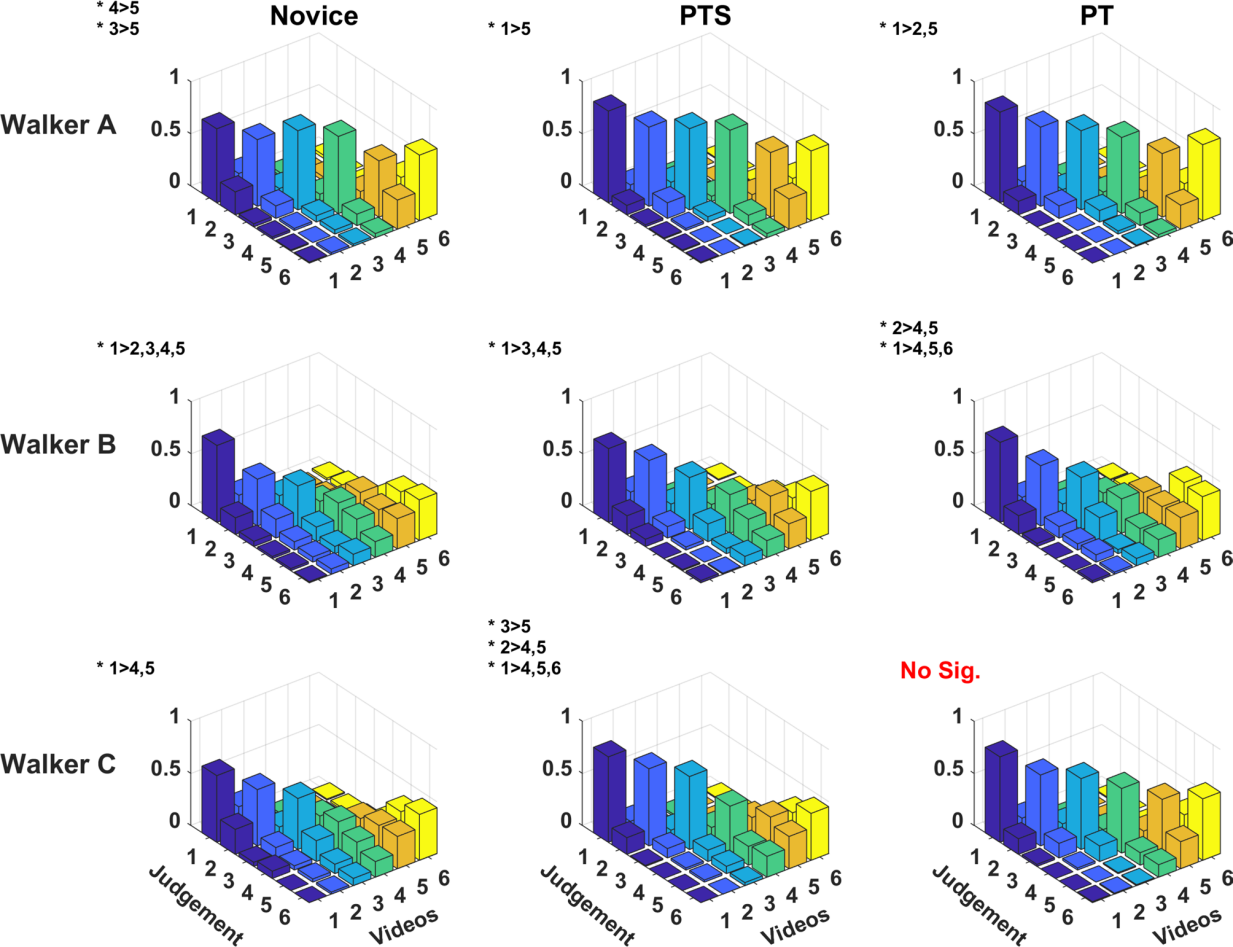
Distribution of judgements in each walker’s video. x-axis indicates the number of presented videos, y-axis indicates the viewers’ judgement to the video, and z-axis indicates the percentage of the occurrences for each event. The diagonal line shows the accuracy rate of the correct judgement. Significant difference between videos is presented on the top—left corner with *. The figures in each row from top to bottom shows the result of Walker A, Walker B and Walker C, respectively. The figures in each column from left to right showed the result of Novice, PTS and PT, respectively.

Figure 3 shows the distribution of judgment accuracy for each walker’s videos per group and the significant differences in accuracy. In seven out of the nine distribution of judgements, the video comparisons showed that the accuracy rate on video 1 (100% amputee’s walking) was significantly higher than the videos 4 or 5 (mixture 40% and 20% amputee walking) (*p*’s < 0.045). However, this trend was not significant for PT on Walker C’s videos and for Novices on Walker A’s videos.

#### Absolute error

To assess the magnitude of misjudgment, the absolute error between viewers’ judgment and correct answers was calculated. We found that most misjudgments occurred between similar videos (e.g., 20% with 40% videos) and Walker B’s video set was the one with more misjudgments. Figure 2B shows the result of absolute error for each group in terms of each walker. The permutation test revealed only a main effect for walker (Walker: *p* = 0.002; Groups: *p* = 0.063; Interaction: *p* = 0.899). The pairwise comparison indicated that Walker B’s video set was more misjudged compared with Walker A’s video set (*p* = 0.002) with no other differences between videos.

### Gait difference between amputee and control walker

#### Variance accounted for (VAF) by PCs (%)

Table 1 shows the VAF for the first four PCs and their accumulated variance. Four PCs could explain 92.98, 96.51 and 97.10% of the data variance in Walker A, B, and C, respectively. For the variance contributed by each PC, PC1 accounted for more than 60% of the total variances for all walkers except for Walker A (38.30%). In Walker A, the first PC was less dominant, which results in higher variance accounted for by PC2 (34.63%) compared to other amputee walkers.

**Table 1:** The percentage of variance that explained by each PC (%) for each walker.

	Walker A	Walker B	Walker C	Control
PC1 (%)	38.30	61.84	72.56	86.02
PC2 (%)	34.63	21.04	13.95	9.15
PC3 (%)	16.85	10.54	6.86	3.38
PC4 (%)	3.20	3.09	3.73	1.45
Total (%)	92.98	96.51	97.1	100

For each walker, four PCs take into account > 90% total variance even for the K3 amputee Walker A.

#### Joints position variance and asymmetry

**Figure 4:**
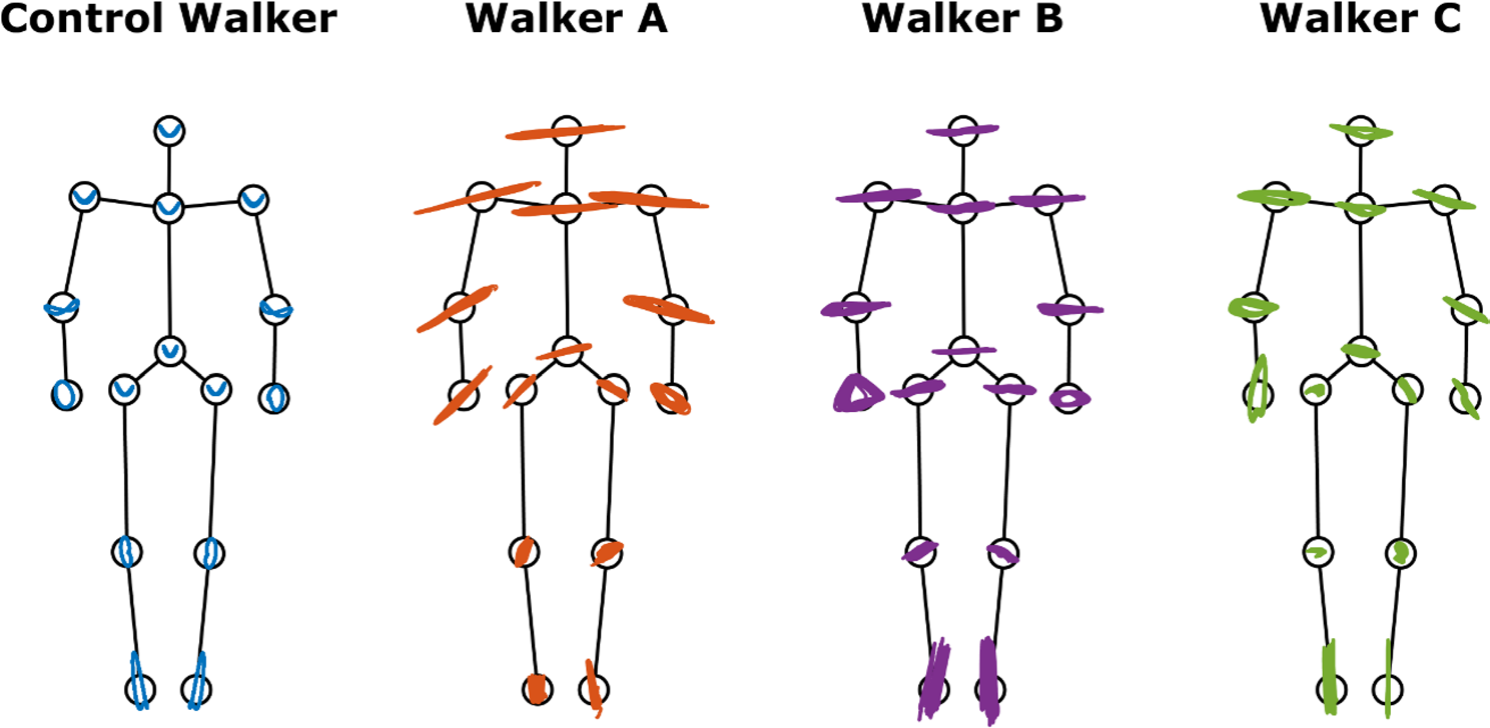
Reconstructed joint trajectories. The joint trajectories were reconstructed from the summation of PC1–PC4 and showed the frontal view of each walkers. The circles represent position of the 15 markers: head, shoulders, elbows, wrists, pelvis, hip, knees, ankles, and clavicle. This figure shows the walkers’ joints variation direction and range of motion that was used to synthesize the video set.

Figure 4 shows the joints trajectories that were reconstructed by the summation of the first 4 PCs. The figure provides the joint position variance and direction across amputees and control walkers. The amputee joint position variance for all amputees was larger than Control Walker and the variation was especially pronounced for Walker A. Besides, Walker C had the largest asymmetry angle compared to the other amputees (see Table 2).

**Table 2:** Degree of gait symmetry for each reconstructed joint trajectory.

	Shoulder	Elbow	Wrist	Hip	Knee	Ankle	Total
Walker A	10.13	17.22	7.95	14.19	64.09	3.32	116.89
Walker B	2.32	6.35	39.18	13.84	1.06	11.51	74.24
Walker C	12.73	25.23	16.70	57.10	69.97	2.19	183.93

Values closer to zero refer to symmetrical pattern.

#### Similarities and differences between Amputees and Control PCs

Table 3 shows the result of normalized dot product for all paired PCs. We highlighted values that were greater than 0.8 that indicated the pattern represented by two PCs were similar. An example of reconstructed movement pattern from similar and not similar PC pairs is shown in Supplementary Movie B. Walker A’s PC2 and PC4 were similar to Control Walker’s PCs accounting for 37.83% data variance. Walker B’s PC1, PC3 and PC4 and Walker C’s PC1, PC2 and PC4 were similar to control walker accounting for 75.47% and 89.88% of data variance, respectively. These results show that, overall, Walker A was the easiest to differentiate from the control walker considering its overall pattern of variation.

**Table 3:** Normalized dot product: paired PCs across normal and amputee walkers.

	Walker A				Walker B				Walker C			
	PC1	PC2	PC3	PC4	PC1	PC2	PC3	PC4	PC1	PC2	PC3	PC4
**Control walker**												
PC1	0.52	**0.81**	0.21	0.01	**0.98**	0.14	0.03	0.02	**0.98**	0.03	0.11	0.01
PC2	0.35	0.23	0.21	0.04	0.03	0.17	**0.85**	0.01	0.01	**0.85**	0.34	0.09
PC3	0.06	0.03	0.05	**0.82**	0.01	0.07	0.04	**0.95**	0.01	0.07	0.03	**0.97**
PC4	0.08	0.07	0.23	0.03	0.04	0.23	0.12	0.10	0.04	0.23	0.20	0.03

We calculated the normalized dot product for all pairing PC between amputee walker and control walker. The paired PCs with value higher than 0.8 are bold.

**Figure 5:**
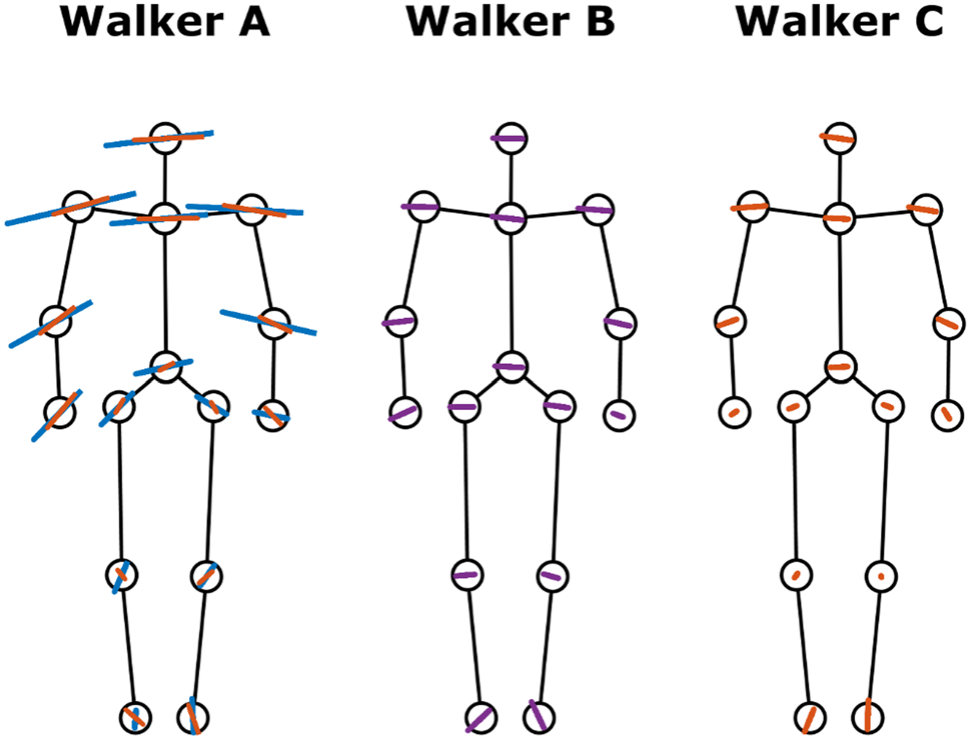
Differentiate joint position variance. The stick figures of walkers are facing to the viewers. The blue, purple, and red lines indicate the reconstructed joint trajectories of the dissimilar PCs in respected to amputee walker’s PC1, PC2, and PC3, respectively.

Figure 5 shows the reconstructed joint trajectories for the PCs that are dissimilar to the control walker. We found that Walker A’s dissimilar PCs (PC1) presented a larger upper body sway in the medial–lateral direction and such sway pattern did not covary with any control walker’s PCs. For the amputee walker B and C, the dissimilar PCs shows a small range of motion.

## Discussion

It is believed that long-lasting changes in perception can be achieved through experience dependent learning^29^. Athletes are sensitive to the opponent’s serving posture to anticipate ball landing location^30^; musicians are better at discriminating pitch and musical style/genre^31,32^. Hence, we expected that PT, given their training and experiences, would show better performance than PTS and Novices in rating gait abnormality. We found that PT and PTS were not significantly different from each other and that both were more accurate than the Novice group (Fig. 2). In some sense, the major differences that we found could be attributed to the directed experience provided in graduate coursework and clinical exposure. Because greater learning changes may occur at the beginning of practice^33^, it appears that if the PTS can attend to the information that is beneficial to differentiate gait impairment, they can perform better than Novices and reach comparable levels of performance as PT rather quickly with respect to OGA.

However, we found minor differences in the accuracy pattern between PT and PTS. The PT’s experience was evident in evaluating the results of Walker C’s videos set (Fig. 3), where the PT group seemed to accurately differentiate across the whole spectrum from normal to abnormal walking patterns. It is of interest to know what gait feature led the PT group to a better response in the videos. As noted, Walker C’s gait was the closest to the control walker of all the amputee walkers (89.88% similarity rate), although with more detailed analyses, a larger deviation from gait symmetry was observed (Fig. 4 and Table 2). Thus, we postulate that the small percentage of deviation on gait symmetry was prominent for PT to pick up.

Gait asymmetry is associated with many pathologies (e.g., hemiplegia, neuropathies, myopathic gait, etc.), has been used in clinical gait assessment, and is largely discussed in the literature^34–36^. Also, some observational gait formularies and gait analysis systems are directly related to gait symmetry (e.g., Brunnstrom’s gait analysis form or GAITRite system). As the PT group has more practical experience with observing and assessing gait symmetry, they likely have learned to promptly attend to this particular feature. This is one potential reason why the PT group may have performed better at discriminating between different levels of gait impairment.

We believe that the greatest outcome from PTs experience is the ability to infer the appropriate causes and plan interventions based on the observed specific pattern. It might be possible that the main differences between PT and PTS are this later part of OGA: the diagnosis. Hence, we might be able to see larger differences between PT and PTS if they were asked to perform the diagnosis. A future study can consider this to fully test our proposed method.

Furthermore, we also found that the relation between actual change of gait impairment and the perceived change is nonlinear (Fig. 3). The gait variation between sequentially synthesized walkers-videos was fixed by a 20% interval, but our result showed a tendency of individuals to perform better at differentiating videos that were close to the amputee-gait pattern. Such nonlinear relations can be traced back as far as Fullerton and Cattell’s law (1892)^37^ or Steven’s power law^38^. These were demonstrated on the nonlinear relation between discrimination of and actual increase in magnitude of force, brightness, and temperature. Our study demonstrated a similar nonlinear relation. This is of interest as it identifies a region on the spectrum of gait “quality” that requires further practice to be differentiated. Then, this region, closer to normal walking (representing more subtle gait deviations), should be emphasized during PT education.

In this study, PCA-based movement analysis, as opposed to classical gait analysis, quantified differences in the coordination/covariation between variables. The original marker positions represent directly measured information without any pre-selection of variables or definition of arbitrary axes to define the motion of the joint. The marker positions facilitate the creation of stick figures to visualize the differences in the movement patterns between amputee and control walker (Fig. 4 and 5) and thus provide a holistic viewpoint when interpreting the results. This approach has been used in gait analysis^20,24–26,39^. Some of these studies reported lower explained variance in the first 4 PCs^24,25^ when analyzing healthy individuals compared to our results. This could be due to the normalization process that minimizes the variability of the input variables. Such procedure was applied given the intent to compare different tasks (walking and running)^24^ or include different types of input variables (e.g., ground reaction force and positional data)^26^. Nevertheless, the explained variance in our study (~ 90%) is similar to others that applied the same PCA procedure^20,39^.

PCA quantified the major dissimilar features between amputee and control walkers that allow individuals to perceive and differentiate walker patterns. The quantified features represent the magnitude of medial–lateral body sway, degree of gait asymmetry, and percentage of gait deviation.

We found that the largest spatial deviation is on the medial–lateral body sway for all amputee walkers (Table 3 and Fig. 5). Also, a high degree of spatial asymmetry in gait was presented especially on Walker A and Walker C (Table 2 and Fig. 4). We postulate that these gait features are the perceptual variables that allow individuals to differentiate between walkers. While the amputee gait was weighted and proportionally presented, the features that contain the major differences would stand out for individuals to attend to rather than other subtle differences. Moreover, gait asymmetry and large medial–lateral body sway are two of the signature features for unilateral transfemoral amputees to compensate pelvic obliquity and push-off deficiency^16,17,40^. Since PT and PTS have been taught these compensatory mechanisms in gait, they might attend to these features to evaluate gait.

By identifying similar PCs between amputee and control walker, we could quantify the amputee’s gait deviation in percentage. This percentage provides a general idea about the qualitative difference in gait and seems to align with the accuracy rate—the lower percentage of gait similarity (Walker A: 38.3%) resulted in a higher accuracy rate (> 67%). We expected Walker C’s pattern to be the hardest to differentiate due to the high gait similarity (15% more than Walker B). However, we found that the Walker B’s video set was the one with largest misjudgment (absolute error > 1.38) and lower accuracy. Even though Walker B is more dissimilar than the control walker than Walker C, when considering the overall pattern of his motion, gait asymmetry might play a stronger role in OGA.

Biological motion has been used to find kinematic information that is beneficial to identify gender^2,20^ or differentiate movement patterns^3,41^. Even though biological motion presents the motion of joints center, it is not that individuals are based on the motion of the joint centers per se in biological motion. The point-light display elicits perception of the kinematic features that inform what is happening (i.e., someone is walking) and features of the walker (e.g., body type, emotions, speed, symmetry, etc.). Thus, the relation to the clinical routine lies in that these kinematic features are also present in the real life situation and can be based on the motion of other parts of the body. We used the joint center motions to analyze the data but, here we were concerned with the kinematic features that could be extracted from their motion. Thus, although the usage of point-light display seems far removed from the clinical settings, it preserves its main features.

The present method, based on biological motion, can be a useful tool for training and evaluating individual’s capability of observation gait analysis. The method allows the viewer to direct attention to the kinematic information distributed from the motion of major joints, and hence the training/evaluation is purely on viewer’s perception of motion instead of other confounding sources of information. The biological motion model is also customizable, allowing manipulation of walking speed, body structure and different levels of gait impairment. Point-light displays motion also simplify the information for visual attention. Thus, a teacher can control/emphasize the kinematic information that they would like students to attend to. We believe this has large potential to improve the training of OGA, especially in the early stage of learning.

Understanding the method that PT use to identify kinematics features could then be incorporated into machine-learning based gait diagnosis, the design of automatic powered prosthesis tuning, or other new technologies in smart health applications. Our research group has been interested in the design of automatic tuning methods for robotic prostheses for lower limb amputees^42,43^ and this has been a formidable challenge. In the clinics, clinicians must manually and heuristically tune the control parameters of robotic prostheses for individual amputees based on observed gait patterns in order to provide personalized gait assistance. This procedure continues until the gait pattern “looks good”, which is burdensome, inaccurate, and time consuming. One of our approaches was to design a cyber expert system that mimicked the decision-making process of clinicians^42^. However, one challenge for this concept and application was the difficulty in knowing the tuning goal of clinicians. The results and methods of this study might help fill this knowledge gap. We showed that clinical professionals seem to attend to gait asymmetry and mediolateral body sway. These features could facilitate the fine-tuning of gait and could be used as the optimization goal for automated tuning machines in the future^42,44^.

This study has a few limitations arising from its novelty. First, we presented the biological motion only in the frontal plane. Considering we did not have prior knowledge on this matter, we chose the frontal plane based on our pilot results that demonstrated a higher performance for Novices. We then considered this plane to be more “informative” and/or easier to result in differentiation between rater groups when judging videos in a short period of time. Nevertheless, this was not problematic as we distinguished experience levels reasonably well. Future studies could investigate another viewing plane to see whether our results are generalizable. Second, our analyses (i.e., PCA) were based on the motion of markers rather than joint angles—which can be another source of features perceived by our raters. Additionally, we made arbitrary choices related to video playing time and the size of the viewing figure. This was based on the requirement to display the videos with a comfortable view and to complete the data collection within PT/PTS’s break hours. It could be that with longer time for gait judgement, PT might demonstrate a better performance as perception of some features might take longer periods of time—a matter for future studies. Finally, it is possible that the gait demonstrated by the three amputee walkers in this study might not be generalizable to the overall amputee population. However, the large ML body sway and gait asymmetry demonstrated by the three amputee walkers are common features of amputee gait. Moreover, the three walkers were sufficiently different to highlight gait features and differentiate the raters’ experience level.

## Methods

### Participants

#### Amputee walker

We recruited three males with unilateral transfemoral amputation (age: 51.3 ± 18.7 years) so that we could create the biological motion videos based on their gait patterns. All amputee participants wore a prosthesis daily for at least 8 or more hours, were able to walk independently without assistance (i.e. K-level 3 or 4) and had previous experience with treadmill walking. All of them reported regularly participation in sports or activities besides walking. The details of the amputees are provided in Table 4.

**Table 4:** Description of amputee subjects.

Walker	K level	Age (year)	Weight (kg)	Height (cm)	Time since amputation (years)	Prescribed prothesis	Side of amputation	Prefer walking speed (m/s)
A	3	65	63	163	7	Symbionic (Össur)	Left	0.7
B	4	63	64	164	17	C-Leg 2 (Ottobock)	Left	1.0
C	4	25	69	180	9	Genium (Ottobock)	Right	1.0

#### Groups of raters

Three groups of raters participated in this study. The PT group consisted of 10 licensed physical therapists (age: 46.2 ± 12.7 years, 5 males,) who had 18.8 ± 13.2 years of experience in treating and performing OGA in clinic. Five PT were currently working with amputees. The PTS group consisted of 10 physical therapy students (age: 24.9 ± 3.4 years, 4 males, 1.7 years in graduate school) who had successfully completed coursework in observational gait analysis and had fieldwork experience working in clinical settings. The Novice group consisted of 10 inexperienced raters (age: 24.3 ± 3.3 years, 5 males). These novice raters had no training in gait analysis/clinical experience.

All participants (amputees and raters) signed an informed written consent form to participate in our protocol—All procedures performed in study was approved by the Institutional Review Board of the University of North Carolina at Chapel Hill. We conducted our study in accordance with the relevant guidelines and regulations.

### Testing biological motion videos and apparatus

#### Data collection

We recorded gait kinematics from the 3 amputee walkers to elaborate the biological motion videos. In the experimental visit, the amputee walkers were asked to walk on a treadmill at a self-selected comfortable speed. For familiarization with the context, participants completed at least 2 min of walking before we started to record the 40 steps (20 full-gait cycles). Participants were not notified when the recording started. We employed the Vicon full body plug-in gait model (40 reflective markers, including sacrum) for the kinematics data collection. VICON motion capture system equipped with 12 high-speed cameras was used for tracking the three-dimensional trajectories of the markers at 100 Hz sample rate (VICON; Oxford, UK).

In order to formulate the biological motion videos, we first computed the location of virtual markers positioned at the major joints or segments of the body (i.e. joints of the ankles, knees, hips, wrists, elbows, shoulders, and in the center of the pelvis, sternum, and head). The locations of these virtual joint markers in the biological motion video matched those used for the control walker. For the control walker (nonimpaired gait), the 15 joint marker trajectories were reconstructed using a human walking model made by Troje^20^ from 25 able-bodied men and 25 able-bodied women. This model was derived using PCA and a sine function by having a classifier distinguishing male and female features in walking. It can generate average walking pattern by means of weighing the gender scale. The male structure (weighting as 1) was used for the Control walker to fit the gender of the amputee walkers.

Equation 1 describes the model of the control walker:

1 $$ {\mathbf{C}}\left( {\mathbf{t}} \right) = {\mathbf{c}}_{0} + { }{\mathbf{c}}_{1} \sin \left( {{{\varvec{\upomega}}}{\text{t}}} \right) + {\mathbf{c}}_{2} \cos \left( {{{\varvec{\upomega}}}{\text{t}}} \right) + {\mathbf{c}}_{3} \sin \left( {2{{\varvec{\upomega}}}{\text{t}}} \right) + {\mathbf{c}}_{4} \cos \left( {{{\varvec{\upomega}}}{\text{t}}} \right) $$

where ${\mathbf{C}}$(t) represents the 45-dimensional vector describing the 15 joints position in 3D of the control walker. The joints positions were reconstructed based on a linear combination of the average posture ${\mathbf{C}}_{0}$ plus the sum of the first four PCs at given time **t**. ${\mathbf{C}}_{{\varvec{i}}}$ represents the first four eigenpostures with a male structure and ${{\varvec{\upomega}}}$ is the fundamental frequency of the walking.

#### Synthesizing biological motion videos

The biological motion videos presented a range of scaled gait abnormalities to test the accuracy of raters. We built three models using PCA, each of which was derived based on the gait pattern of one amputee walker and the control walker. Each model described the 3D motion of the 15 joints and was used to synthesize six biological motion videos that scaled gait abnormality by varying the features from the original amputee gait (0%) to the control walker (100%) in steps of 20%. Thus, the level of gait abnormality within the 6 videos was varied from most abnormal (the amputee gait) to most normal (control walker’s gait) for raters to sequence. To synthesize each biological motion video, first, the joint trajectories of amputee walkers were low-pass filtered using a fourth order zero-lag Butterworth filter set at 6 Hz. We time-normalized the number of data samples within each gait cycle for the joint trajectory data of each amputee walker and the control walker. Then, PCA was performed to extract the eigenvalues, eigenvectors and scores for each amputee walkers:

2 $$ {\mathbf{A}}_{{\varvec{j}}} \left( {\text{t}} \right) = {\mathbf{C}}_{0} + { }\mathop \sum \limits_{{{\varvec{i}} = 1}}^{4} {\mathbf{S}}_{{{\varvec{ij}}}} {\mathbf{P}}_{{{\varvec{ij}}}} \left( t \right) $$

where ${\mathbf{A}}_{{\varvec{j}}} \left( {\varvec{t}} \right)$ represents the amputee walker **j**’s joints position in 3D (45-dimensions). The joints positions are the average posture ${\varvec{C}}_{0}$ of the control walker plus a weighted sum of the first four PCs. ${\mathbf{P}}_{{{\varvec{ij}}}}$ denotes the ith principal component for the amputee walker **j** and ${\mathbf{S}}_{{{\varvec{ij}}}}$ denotes the respective scores. The walker’s body structure was normalized to fit the control walker for all synthesized walkers.

To synthesize the gait features ranged from control walker to amputee walker, we combined Amputee PCA model and Control walker model with a weighting term:

3 $$ {\mathbf{B}}_{{{\varvec{jk}}}} = {\mathbf{C}}_{0} \user2{ } + \user2{ }\left( {1 - {{\varvec{\upalpha}}}_{{\varvec{k}}} } \right){\mathbf{A}}_{{\varvec{j}}} \left( {\text{t}} \right) + {{\varvec{\upalpha}}}_{{\varvec{k}}} {\mathbf{C}}\left( {\text{t}} \right) $$

where ${\mathbf{B}}_{{{\varvec{jk}}}}$ represents the joints positions that are synthesized between the amputee walker **j** and control walker by weighting ${{\varvec{\upalpha}}}_{{\varvec{k}}}$. k denotes the kth ${{\varvec{\upalpha}}}_{{\varvec{k}}}$ that was used to generate the testing video.

We generated the six biological motion videos from each model ${\mathbf{B}}_{{{\varvec{jk}}}}$ (Eq. 3). The six videos displayed the synthesized biological motion scaled gait abnormality by varying ${{\varvec{\upalpha}}}_{{\varvec{k}}}$ from 0 to 100% (in steps of 20%). In this way, the video set generated from the biological model contained two videos with the real gait of amputee (${{\varvec{\upalpha}}}_{{\varvec{k}}} = 0\user2{\% }$) and control walker (${{\varvec{\upalpha}}}_{{\varvec{k}}} = 100\user2{\% }$) and videos in between had decreasing/increasing weighing from the amputee/control walker.

All biological motion videos were presented in 2D frontal view (walking toward the raters) with gait frequency at 1 Hz. We chose this view given that Novice participants, in a pilot test, preferred and performed better on the frontal view. In all biological motion videos, the joint positions of the synthesized walker appeared as yellow dots on a black background. The videos were generated using MATLAB (MathWorks, Natick, MA). A sample testing video set generated from amputee Walk A is presented in Supplementary Movie A.

#### Procedure for gait rating

The video sets were shown at raters’ preferred distance and height and displayed on a DELL 27-in. LED color monitor with 1920X1080 resolution at 60 Hz. For each trial, six biological motion videos generated from one of the amputees simultaneously arranged into 2 row and 3 columns in a random order were presented for 23 s (20 gait cycles). The goal for the raters were to rank the videos from 1 to 6 based on their perceived gait abnormality (1-most abnormal, 6-most normal). Raters received no feedback on their answers. After raters completed their answer, the next set of videos was displayed. All three videos sets were tested and presented for 10 times in a random order (30 trials: 3 video sets*10 repetitions). The total testing time was about 40 min. A practice trial (Walker A video set) was presented for raters to familiarize the task.

### Data analysis and statistics

#### Raters’ judgement

To estimate the participants abilities to judge walking patterns, we calculated each rater’s (1) accuracy rate and (2) absolute error in judging each Walker’s videos. The accuracy rate evaluates the rater’s ability to differentiate abnormal gait and the absolute error measures the magnitude of misjudgment. We expect that the experience level will determine the accuracy rate and absolute error (PT will be the best group, followed by PTS and, then, Novices).

4 $$ {\text{Accuracy}}\,{\text{rate}}\,{\text{in}}\,{\text{judging}}\,{\text{walker}} = \frac{counts\,of\,correct\,judgement}{{6 \times 10 \left( {videos \times repeated\, trials} \right)}} $$

5 $$ {\text{Absolute}}\,{\text{error}}\,{\text{in}}\,{\text{judging}}\,{\text{walker}} = \frac{{\sum \left| {judgement - correct\,answer} \right|}}{{6 \times 10 \left( {videos \times repeated\,trials} \right)}} $$

#### Decomposing kinematics features using PCA

To investigate what are the key kinematics features that raters perceived to identify gait deviations, we quantified the gait pattern difference between amputee walkers and the control walker based on the extracted patterns from PCA.

#### Variance accounted for by PCs (%) and joints position variance

To synthesize the point-light walkers, we utilized the first four dominant Principal Components (PC) of the PCA analysis matching the number Troje^20^ used for the control walking model. We reported the variance accounted for (VAF) by each PCs and reconstructed joint position variance to compare the overall PC differences between amputee and control walker.

#### Differentiating similar and dissimilar movement patterns between amputee and control PC

Given the task constraints (all individuals were walking), we expected that amputees shared similarities with the control walker. This would be evident in the PCs structure. Similarly, some PCs would reveal differences referring to the functional limitations of amputee’s motor impairment. To differentiate similar and dissimilar PCs, the normalized dot product was calculated between all possible PC pairs across amputees and control walker.

$$ \cos \theta_{ij} = \frac{{\overset{\lower0.5em\hbox{$\smash{\scriptscriptstyle\rightharpoonup}$}}{{u_{i} }} *\overset{\lower0.5em\hbox{$\smash{\scriptscriptstyle\rightharpoonup}$}}{{v_{j} }} }}{{\overset{\lower0.5em\hbox{$\smash{\scriptscriptstyle\rightharpoonup}$}}{{u_{i} }} \overset{\lower0.5em\hbox{$\smash{\scriptscriptstyle\rightharpoonup}$}}{{v_{j} }} }} $$

where $$\overset{\lower0.5em\hbox{$\smash{\scriptscriptstyle\rightharpoonup}$}}{{u_{i} }}$$ and $$\overset{\lower0.5em\hbox{$\smash{\scriptscriptstyle\rightharpoonup}$}}{{v_{j} }}$$ are the eigenvector corresponding to amputee and control walker’s PC_i_ and PC_j_. If the absolute value of $$\cos \theta_{ij}$$ is 1, the paired PC_ij_ overlap, meaning that position of joints motion in both PC_i_ and PC_j_ covary in the same manner and demonstrated similar movement pattern. If the value is 0, the covariation of joint motion in PC_i_ and PC_j_ are orthogonal to each other and are not related.

#### Determining the degree of gait symmetry

We calculated the main eigenvector on the reconstructed trajectories of shoulder, elbow, wrist, hip, knee and ankles on both right and left limbs, and measured the angle between eigenvector and horizontal axis. For the limbs on the right and left side, the angle was defined on the 1st and 3rd quadrant, respectively. The difference between angles of the paired contralateral joints was used to determine the degree of gait symmetry. Values closer to zero refer to symmetrical pattern.

### Statistical test

We performed permutation tests comparing accuracy rate and absolute error in terms of rater *groups and walkers* (3*3) to examine if experience leads to a better judgment of walkers; and in terms of rater *groups and video* (3*6) was to test if experience leads to better sensitivity to different gait patterns. A permutation test was applied based on Manly’s approach^45^ for factorial 2-way ANOVA design. By using a permutation test, we can bypass assumptions of continuity, normality, and homoscedasticity still taking advantage of the full sample information^46^ providing that the judgement rates and absolute errors are not continuous numbers. The permutation multiple comparison was utilized based on one-sample t-test for group difference and paired t-test for walker/videos. T-max method was used for adjusting the *p*-values of each variable for multiple comparison^47^. The significant level was set at α = 0.05.

## Supplementary information

Supplementary Video Control_PC1.

Supplementary Video Control_PC2.

Supplementary Video Control_PC3.

Supplementary Video Control_PC4.

Movie A - testing trial.

Movie B - similar & dissimilar PCs.

Supplementary Video Walker A_PC1.

Supplementary Video Walker A_PC2.

Supplementary Video Walker A_PC3.

Supplementary Video Walker A_PC4.

Supplementary Video Walker B_PC1.

Supplementary Video Walker B_PC2.

Supplementary Video Walker B_PC3.

Supplementary Video Walker B_PC4.

Supplementary Video Walker C_PC1.

Supplementary Video Walker C_PC2.

Supplementary Video Walker C_PC3.

Supplementary Video Walker C_PC4.
